# Quality Comparison of Apios Starch Gel and Whole Apios Gel and Effect of Oil Addition on Retrogradation Inhibition in Whole Apios Gel

**DOI:** 10.1002/fsn3.71496

**Published:** 2026-02-10

**Authors:** Soo Hyun Kim, Ju Hong Park, Nami Joo

**Affiliations:** ^1^ Department of Food and Nutrition Sookmyung Women's University Seoul Republic of Korea; ^2^ Department of Convergence IT Engineering Pohang University of Science and Technology (POSTECH) Pohang Republic of Korea

**Keywords:** *Apios americana*, gelled food product, oil addition, retrogradation inhibition, starch

## Abstract

*Apios americana* is an underutilized legume rich in carbohydrates, proteins, and micronutrients, yet research on its potential as a gel‐based food and its retrogradation behavior remains limited. This study aimed to compare the physicochemical and sensory properties of gels prepared from whole Apios and Apios starch, and to investigate the effect of oil addition on retrogradation inhibition in whole Apios gel. Texture profile analysis, syneresis rate measurement, sensory evaluation, Differential Scanning Calorimetry (DSC), and Fourier Transform Infrared Spectroscopy (FTIR) were conducted to analyze gel characteristics and structural changes. Whole Apios gel showed significantly lower syneresis and superior texture acceptability compared with starch gel, receiving the highest overall liking score. Increasing oil content (0%–6%) decreased hardness, improved elasticity, and effectively reduced syneresis. DSC results indicated the lowest enthalpy value at 4% oil addition, demonstrating optimal retrogradation inhibition. FTIR analysis confirmed reduced hydrogen bonding and recrystallization, supporting the stabilizing mechanism of oil incorporation. These findings highlight the functional potential of whole Apios gel as a retrogradation‐resistant gel product and suggest practical applications for value‐added product development utilizing underused crops in the food industry.

## Graphical abstract

Whole Apios gel demonstrated superior quality characteristics compared to Apios starch gel, including lower syneresis and improved sensory preference due to its nutrient‐rich composition. The addition of oil effectively inhibited retrogradation, with 4% oil achieving optimal textural stability, reduced enthalpy change (ΔH), and decreased hydrogen bonding intensity. These findings indicate the potential of whole Apios gel as a functional and stable gelling food product without starch extraction.

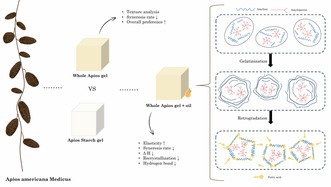

## 1 Introduction

Apios ( *Apios americana* Medicus), also known as groundnut, is a perennial vining tuberous plant in the legume family, indigenous to North America (Li et al. 2021). Apios tubers contain carbohydrates, protein, dietary fiber, calcium, iron, vitamin C, and bioactive compounds such as saponins and isoflavones, which contribute to antioxidant, antihypertensive, and antidiabetic activities (Keneta et al. 2016; Iwai and Matsue 2007; Kim et al. 2021). While numerous studies have focused on the bioactive and physiological functions of Apios, research on its application in food processing and product development remains limited. In particular, the utilization of Apios as a gel‐based food material has not been sufficiently explored.

Starch‐based gels are widely used in traditional and commercial food systems, and their quality characteristics are largely influenced by the amylose–amylopectin ratio, crystalline–amorphous structure, and molecular interactions within the gel matrix (Lan et al. 2024). However, a major limitation of starch gel applications is retrogradation, a process in which gelatinized starch chains gradually reassociate and recrystallize during storage, leading to increased hardness, syneresis, and reduced consumer acceptability. To address this problem, formulation strategies using lipids, proteins, and sugars have been explored, as these components can form stabilizing interactions with amylose and amylopectin. In particular, lipid incorporation has been shown to delay recrystallization by forming amylose–lipid complexes that modify molecular packing and reduce retrogradation‐associated enthalpy (Zhou et al. 2025; Castro‐Campos et al. 2024).

In conventional gel production, starch is typically extracted through wet milling, sedimentation, and drying processes, which often result in the loss of valuable nutrients and generate substantial by‐products. Although recent studies have investigated advanced starch extraction technologies, research utilizing whole tuber materials without starch isolation remains limited. Given that whole Apios contains proteins, dietary fiber, minerals, and vitamins in addition to carbohydrates (Li et al. 2021), producing gel directly from whole Apios may offer nutritional and environmental advantages compared to starch‐isolated gels, as also suggested by our preliminary observations.

Therefore, this study aimed to compare the physicochemical and sensory properties of gels prepared using whole Apios and extracted Apios starch, and to investigate the effect of oil addition on retrogradation inhibition in whole Apios gel during storage. Texture analysis, syneresis measurement, sensory evaluation, Differential Scanning Calorimetry (DSC), and Fourier Transform Infrared Spectroscopy (FTIR) were employed to elucidate structural and functional changes. This research provides insights into the development of functional gel products utilizing whole tuber materials and supports the sustainable use of underutilized crops.

## 2 Materials and Methods

### 2.1 Materials

The Apios ( *A. americana* ) utilized in this study was harvested in March 2025 from Jinju, Gyeongsangnam‐do, South Gyeongsang Province, Korea. It was selected for its softness and firmness, with no decay or surface wounds, and stored in a refrigerator (WOOSUNG, WSM‐830R) at 4°C until use. The storage periods of 1 day, 3 days, 7 days, and 14 days were chosen for the experiment to observe the progression of retrogradation and quality changes in the gel over time. These time intervals are commonly used in food science research to capture both short‐term and long‐term changes in texture, syneresis, and structural properties. By including these specific durations, the study aimed to analyze the effects of oil addition on retrogradation inhibition during different stages of storage, providing a comprehensive understanding of how oil impacts the gel's stability and quality over time.

### 2.2 Quality Characteristics of Whole Apios Gel and Apios Starch Gel

#### 2.2.1 Preparation of Starch

**FIGURE 1:**
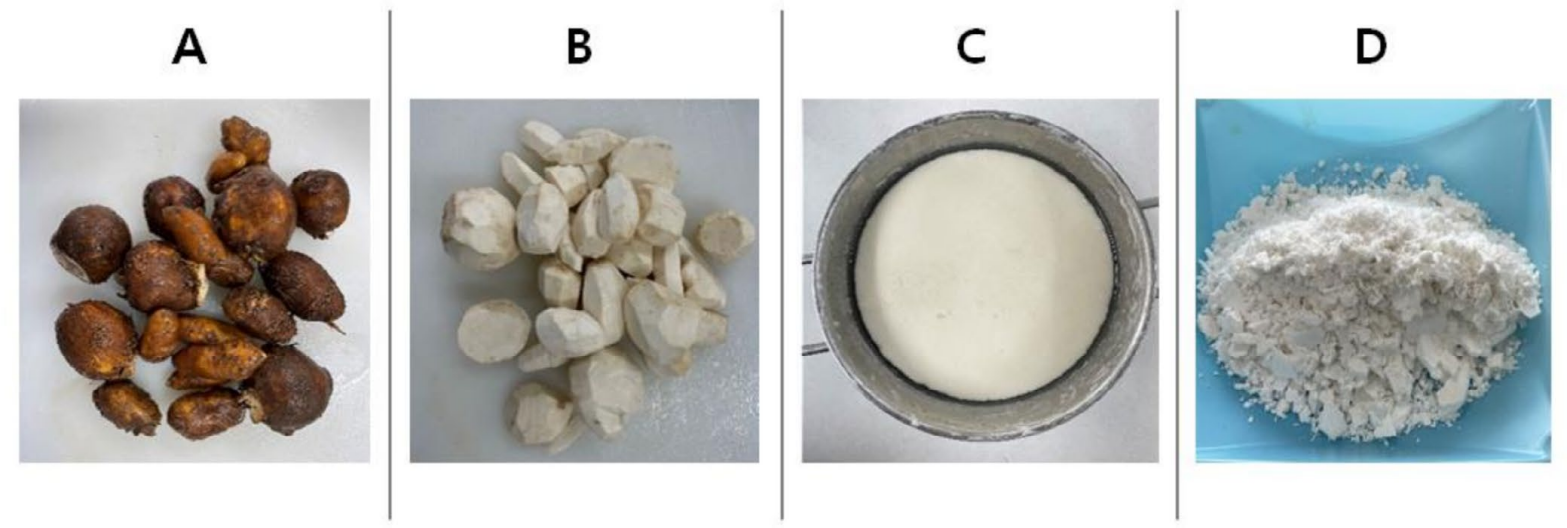
Visual representation of Apios starch extraction process. (A) Raw Apios tubers before washing and peeling. (B) Peeled Apios tubers. (C) Starch sedimentation following grinding and filtration. (D) Dried Apios starch powder after dehydration.

Peeled Apios tubers (300 g) were homogenized in a blender with 1 L of distilled water, and the resulting slurry was filtered through a muslin cloth to collect the starch suspension. The suspension was allowed to precipitate for 2 h with an additional 1 L of water. The precipitated starch was stored at 4°C for 24 h and subsequently dried under sunlight, after which excess moisture was removed (Alam et al. 2020). To visually support the starch extraction procedure, Figure 1 presents original photographs showing the key preparation steps: (A) raw Apios tubers before washing and peeling, (B) peeled Apios tubers, (C) starch extraction and sedimentation, and (D) dried Apios starch powder after dehydration.

#### 2.2.2 Manufacturing of Whole Apios Gel and Apios Starch Gel

Apios and water were mixed in a blender and brought to a boil for 2 min at intensity 7 on an induction cooker (SK Magic, IHRB 3200), followed by cooking for 3 min for whole Apios gel and 2 min for Apios starch gel at intensity 5. The gel was then placed in a 15 × 10 × 8 cm container and allowed to cool for 4 h. The ratio of Apios to water was set at 1:6 for whole Apios gel and 1:5 for Apios starch gel, reflecting the different properties of whole Apios and Apios starch (Nam and Yoo 2022).

#### 2.2.3 Analysis of Texture Properties

The texture of the gel was measured in triplicate using a texture analyzer (TA‐XT Express v2.1, Stable Micro Systems Products London, UK) following the protocol by Lan et al. (2024). It involved the TPA test, a two‐bite test measuring hardness, adhesiveness, elasticity, chewiness, gumminess, and cohesiveness. Test conditions included a pretest speed of 3.0 mm/s, test speed of 3.0 mm/s, posttest speed of 3.0 mm/s, test duration of 2.0 s, trigger force of 1.0 N, and using Texture Profile Analysis (TPA) type. The tested sample dimensions were 1 cm in width, length, and height.

#### 2.2.4 Analysis of Syneresis

The method for measuring the water retention of gel followed the procedure by Nam and Yoo (2022). Post‐preparation, 100 g of gel was placed in a lidded container and stored at 4°C for observations and measurements on days 1, 3, 7, and 14. Experiments were conducted in triplicate and results were presented as average values.

$$\text{Syneresis rate}\;\left(\%\right)=\left\{\text{weight of liquid separated}\;\left(g\right)/\text{weight of}\;\mathrm{gel}\;\left(g\right)\right\}\times 100$$

#### 2.2.5 Sensory Evaluation Test

The sensory evaluation of the two types of gel was conducted with approval from the Institutional Review Board of Sookmyung Women's University (IRB No. SMWU‐2402‐HR‐123). A total of 15 trained graduate students from the Department of Food and Nutrition, who had prior experience with sensory evaluation through coursework and laboratory training, participated as panelists. The evaluation was performed in the Sensory Analysis Laboratory at Sookmyung Women's University under controlled lighting and temperature conditions. Each sample was labeled with a randomized three‐digit code, and panelists rinsed their mouths with water between evaluations to minimize carryover effects. Sensory attributes including flavor, taste, color, elasticity, and overall preference were assessed using a 7‐point scale.

### 2.3 Quality Characteristics of Whole Apios Gel Depending on Oil Content

#### 2.3.1 Manufacturing Whole Apios Gel With Added Oil

After blending the Apios with water, the suspension was transferred to a saucepan, placed on an induction (SK Magic, IHRB 3200), and heated at intensity 7 until a gel formed. Then, oil (HaePyo Canola Oil, Seoul, South Korea) was added according to the concentration (0%, 2%, 4%, and 6%), the intensity was reduced to 5, and the mixture was heated until completion. The final gel was placed in a 15 × 10 × 8 cm container to cool for 30 min, then covered and left to harden at room temperature for 4 h before use in the experiment (Nam and Yoo 2022).

#### 2.3.2 Retrogradation Analysis by Texture Properties

The texture of whole Apios gel with added oil was measured using the same method as the texture measurement of Apios gel.

#### 2.3.3 Retrogradation Analysis by Syneresis

The syneresis of whole Apios gel with added oil was measured using the same method as the syneresis measurement of Apios gel.

#### 2.3.4 Retrogradation Analysis by Differential Scanning Calorimetry (DSC)

Retrogradation properties of the Apios gel were analyzed by measuring melt temperature and enthalpy using Differential Scanning Calorimetry (DSC). Gel samples containing different oil concentrations (0%, 2%, 4%, and 6%) were stored at 4°C for 1, 3, 7, and 14 days. After storage, samples were freeze‐dried at −70°C for 72 h using a Bondiro MCFD 8508 Freeze Dryer (Ilsin, Seoul, Korea) and subsequently ground into powder with a Hanil Electric grinder (HMF‐3100S, Seoul, Korea). Freeze‐drying was performed to standardize moisture content across samples and ensure consistency in thermal analysis conditions. For DSC measurements, 2.0 ± 0.3 mg of powdered sample was weighed into an aluminum pan and mixed with 8.0 ± 0.5 mg of distilled water to achieve a controlled sample‐to‐water ratio of 1:4. Thermal transitions were analyzed using a Simultaneous Thermal Analyzer (SDT 650, DSC2500) from 20°C to 120°C at a heating rate of 10°C/min, and onset temperature (To), peak temperature (Tp), completion temperature (Tc), and enthalpy change (ΔH) were recorded based on the DSC thermograms (Wang et al. 2018).

#### 2.3.5 Retrogradation Analysis by Fourier Transform Infrared Spectrophotometer (FTIR)

Fourier transform infrared spectrophotometry (FTIR) was used to measure the retrogradation properties of whole Apios gel. Whole Apios gel with different oil contents were stored in a refrigerator (4°C) for different storage periods (1, 3, 7, and 14 days), freeze‐dried at −70°C for 72 h in a freeze‐dryer (Bondiro MCFD 8508 Freeze Dryer Ilsin, Seoul, Korea), and then ground in a grinder (Hanil Electric, HMF‐3100S, Seoul, Korea) to powder on a 100‐mesh standard mesh sieve and used as samples. ATR‐FTIR (Thermo Fisher Scientific, Nicolet IS50) was utilized, with a scan range of 800–4000 cm^−1^ and an average of 32 scans (Wang et al. 2018).

### 2.4 Statistical Analysis

Statistical analyses were conducted using SPSS statistics (ver 26. IBM Co., Armory, NY, USA) and are presented as means and standard deviations. The *t*‐test and one‐way ANOVA were used to assess differences between each sample, and if significant differences were found at the *p* < 0.05 level, Duncan's multiple range test was subsequently performed for post hoc analysis.

## 3 Result and Discussion

### 3.1 Quality Characteristics of Whole Apios Gel and Apios Starch Gel

#### 3.1.1 Texture Properties

The textural properties (hardness, adhesiveness, elasticity, chewiness, gumminess, and cohesiveness) of whole Apios gel and Apios starch gel are presented in Table 1. The hardness (*p* < 0.05) and gumminess (*p* < 0.01) of whole Apios gel were significantly higher than those of Apios starch gel. Whole Apios gel also exhibited greater chewiness, indicating a firmer gel structure. Adhesiveness values were negative in both gels, suggesting that neither gel exhibited strong stickiness to oral surfaces. No significant difference was observed in elasticity between the two gels, indicating comparable elastic behavior.

**TABLE 1:** Texture properties of whole *Apios* gel and *Apios* starch gel.

Mean ± SD			
	Whole Apios gel	Apios starch gel	*t*‐value^1^
Hardness (N)	0.37 ± 0.01	0.34 ± 0.01	4.388*
Adhesiveness (g × s)	−0.04 ± 0.01	−0.03 ± 0.03	−0.735
Elasticity (mm)	0.91 ± 0.05	0.90 ± 0.11	0.242
Chewiness (N × mm)	31.34 ± 2.77	26.78 ± 3.44	1.787
Gumminess (N)	34.38 ± 1.10	29.82 ± 0.94	5.481**
Cohesiveness (%)	0.91 ± 0.01	0.93 ± 0.02	−2.214

^1^ *p < 0.05, **p < 0.01.

The higher hardness, gumminess, and chewiness of whole Apios gel may be attributed to the presence of non‐starch components such as proteins and dietary fiber, which are retained when the whole tuber is used (Li et al. 2021). These components are likely to contribute to the formation of a denser gel network through additional intermolecular interactions. Elasticity, an important attribute of gelled foods affecting mouthfeel and structural resilience, remained sufficient in both gels, supporting their suitability as starch‐based gel substitutes (Nishinari and Fang 2018). Similar observations have been reported in composite gel systems, where non‐starch constituents enhance network density and textural stability (Zhou et al. 2025).

#### 3.1.2 Syneresis Properties

Syneresis of whole Apios gel and Apios starch gel stored at 4°C for 14 days was evaluated at 1, 3, 7, and 14 days, and the results are summarized in Table 2. No syneresis was observed in either gel on day 1. On day 3, whole Apios gel showed a significantly lower syneresis rate (4.83% ± 0.58%) than Apios starch gel (5.87% ± 0.40%) (*p* < 0.05). By day 7, the syneresis rate of Apios starch gel increased markedly to 9.13% ± 0.42%, whereas whole Apios gel maintained a lower value of 5.47% ± 0.15% (*p* < 0.001). A similar trend was observed on day 14, with whole Apios gel consistently exhibiting lower syneresis than starch gel.

**TABLE 2:** Syneresis rate of whole *Apios* gel and *Apios* starch gel.

Mean ± SD				
		Whole Apios gel	Apios starch gel	*t*‐value^1^
Storage period (days)	1	0.00 ± 0.00	0.00 ± 0.00	—
	3	4.83 ± 0.58	5.87 ± 0.40	−4.384*
	7	5.47 ± 0.15	9.13 ± 0.42	−14.321***
	14	4.63 ± 0.23	5.80 ± 0.44	−4.096**

^1^ *p < 0.05, **p < 0.01, ***p < 0.001.

Syneresis reflects the structural stability of a gel and is closely associated with starch retrogradation and network contraction (Mizrahi 2010). Gels with higher amylose content generally exhibit greater syneresis due to weaker water‐holding capacity during recrystallization (Singh et al. 2003). The higher syneresis observed in Apios starch gel is therefore likely attributable to its higher starch concentration and absence of stabilizing non‐starch components.

Reduced syneresis is critical for maintaining the appearance, texture, and microbiological stability of gelled foods (Arab et al. 2023). The consistently lower syneresis rates observed in whole Apios gel indicate enhanced water retention and slower retrogradation progression. These results suggest that whole Apios gel maintains structural integrity during storage and offers superior quality performance compared with Apios starch gel, supporting its potential application as a stable gel‐based food product.

#### 3.1.3 Sensory Evaluation

The sensory evaluation results for whole Apios gel and Apios starch gel are summarized in Table 3. Whole Apios gel received significantly higher scores for flavor (4.67 ± 1.11) and taste (4.60 ± 1.40) compared with Apios starch gel (*p* < 0.05 and *p* < 0.001, respectively). No significant differences were observed in color, elasticity, or overall preference between the two gels, although whole Apios gel showed slightly higher mean values for elasticity and overall liking.

**TABLE 3:** Sensory evaluation of whole *Apios* gel and *Apios* starch gel.

Mean ± SD			
	Whole Apios gel	Apios starch gel	*t*‐value^1^
Flavor	4.67 ± 1.11	3.73 ± 0.96	2.458*
Taste	4.60 ± 1.40	2.47 ± 0.91	4.929***
Color	4.93 ± 1.22	5.07 ± 1.28	−0.292
Elasticity	4.27 ± 1.67	4.00 ± 1.69	0.435
Overall preference	4.33 ± 1.68	3.60 ± 1.24	1.361

^1^ *p < 0.05, ***p < 0.001.

Sensory perception of gelled foods is strongly influenced by textural attributes such as hardness and elasticity, which affect mastication behavior and overall acceptability (Chen 2009). The higher preference scores for flavor and taste in whole Apios gel may be related to the retention of intrinsic compounds present in the whole tuber, as well as its firmer yet elastic texture. These findings indicate that whole Apios gel possesses favorable sensory characteristics and supports further investigation into formulation strategies, including oil addition, to enhance storage stability.

### 3.2 Retrogradation Characteristics of Whole Apios Gel Depending on Oil Content

#### 3.2.1 Texture Properties

**FIGURE 2:**
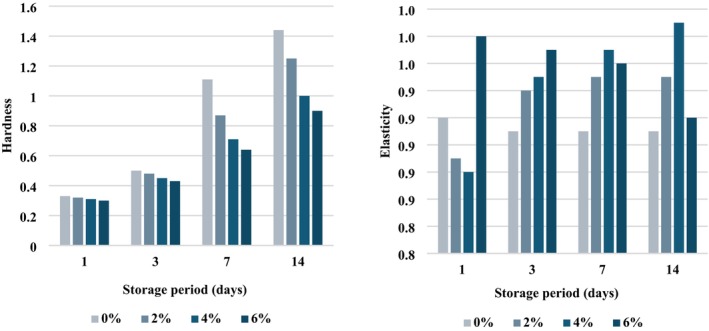
Hardness and elasticity of whole Apios gel based on oil content during storage. This graph depicts the hardness and elasticity of whole Apios gel with 0%, 2%, 4%, and 6% oil added, measured over 1, 3, 7, and 14 days at 4°C.

The texture characteristics of whole Apios gel with different oil contents (0%, 2%, 4%, and 6%) were analyzed during storage (Table 4, Figure 2). Hardness increased significantly during storage across all samples (*p* < 0.001). After 14 days, the hardness of the control gel (0% oil) reached 1.44 ± 0.00 N, whereas the gel containing 4% oil showed a significantly lower hardness of 1.00 ± 0.00 N, indicating a slower progression of structural rigidity and delayed retrogradation. Chewiness decreased substantially with increasing oil content, from 127.62 ± 1.86 N × mm (0% oil) to 96.34 ± 1.99 N × mm (4% oil) on day 14 (*p* < 0.001). Gumminess demonstrated a similar trend, decreasing markedly from 140.21 ± 3.53 N in the control to 97.80 ± 2.02 N in the 4% oil sample. Adhesiveness values became less negative with increased oil concentration (−0.20 ± 0.04 g × s at 0% and −0.02 ± 0.00 g × s at 4%), indicating reduced stickiness. Elasticity remained relatively stable across samples, with slightly higher elasticity in the 4% oil sample (0.99 ± 0.00 mm) compared to the control (0.91 ± 0.02 mm).

**TABLE 4:** Texture properties of whole *Apios* gel depending on oil content during storage.

Mean ± SD						
		Oil (%)				*F*‐value^1^
		0	2	4	6	
Day 1	Hardness (N)	0.34 ± 0.00^a^ ^1^	0.33 ± 0.00^b^	0.31 ± 0.00^c^	0.30 ± 0.00^d^	225.873***
	Adhesiveness (g × s)	−0.02 ± 0.02^a^	−0.04 ± 0.03^a^	−0.04 ± 0.02^a^	−0.02 ± 0.00^a^	1.086
	Elasticity (mm)	0.92 ± 0.06^a^	0.89 ± 0.09^a^	0.88 ± 0.08^a^	0.98 ± 0.01^a^	1.397
	Chewiness (N × mm)	28.84 ± 2.22^a^	27.13 ± 2.59^a^	25.20 ± 2.17^a^	27.18 ± 0.06^a^	1.628
	Gumminess (N)	31.50 ± 0.71^a^	30.65 ± 0.25^a^	28.80 ± 0.59^b^	27.73 ± 0.21^c^	36.732***
	Cohesiveness (%)	0.92 ± 0.02^a^	0.92 ± 0.01^a^	0.90 ± 0.02^a^	0.92 ± 0.01^a^	0.937
Day 3	Hardness (N)	0.50 ± 0.00^a^	0.48 ± 0.00^b^	0.45 ± 0.00^c^	0.43 ± 0.01^d^	67.138***
	Adhesiveness (g × s)	−0.06 ± 0.05^b^	−0.04 ± 0.02^a^	−0.04 ± 0.02^a^	−0.04 ± 0.02^ab^	0.203
	Elasticity (mm)	0.91 ± 0.07^b^	0.94 ± 0.06^ab^	0.95 ± 0.08^a^	0.98 ± 0.01^ab^	0.628
	Chewiness (N × mm)	42.13 ± 3.98^a^	41.92 ± 2.96^a^	40.22 ± 3.19^a^	39.05 ± 1.24^ab^	0.575
	Gumminess (N)	46.51 ± 2.15^a^	44.81 ± 0.64^ab^	42.59 ± 1.67^b^	40.05 ± 1.28^c^	9.918**
	Cohesiveness (%)	0.90 ± 0.04^a^	0.92 ± 0.01^a^	0.92 ± 0.03^a^	0.90 ± 0.01^a^	0.418
Day 7	Hardness (N)	1.11 ± 0.00^a^	0.87 ± 0.00^b^	0.71 ± 0.00^c^	0.64 ± 0.00^d^	14282.499***
	Adhesiveness (g × s)	−0.14 ± 0.08^a^	−0.08 ± 0.04^a^	−0.05 ± 0.04^a^	−0.06 ± 0.05^a^	1.567
	Elasticity (mm)	0.91 ± 0.05^a^	0.95 ± 0.04^a^	0.97 ± 0.04^a^	0.96 ± 0.06^a^	1.020
	Chewiness (N × mm)	95.60 ± 5.22^a^	78.29 ± 4.62^b^	66.71 ± 2.10^c^	60.15 ± 4.17^c^	41.173***
	Gumminess (N)	105.05 ± 4.45^a^	82.79 ± 1.37^b^	68.78 ± 0.99^c^	62.62 ± 0.79^d^	182.537***
	Cohesiveness (%)	0.93 ± 0.04^a^	0.93 ± 0.02^a^	0.95 ± 0.02^a^	0.96 ± 0.01^a^	0.840
Day 14	Hardness (N)	1.44 ± 0.00^a^	1.25 ± 0.01^b^	1.00 ± 0.00^c^	0.90 ± 0.01^d^	4760.906***
	Adhesiveness (g × s)	−0.20 ± 0.04^c^	−0.08 ± 0.03^b^	−0.02 ± 0.00^a^	−0.10 ± 0.03^b^	18.358**
	Elasticity (mm)	0.91 ± 0.02^b^	0.95 ± 0.02^ab^	0.99 ± 0.00^a^	0.92 ± 0.05^b^	5.364*
	Chewiness (N × mm)	127.62 ± 1.86^a^	115.25 ± 4.49^b^	96.34 ± 1.99^c^	77.92 ± 4.78^d^	112.950***
	Gumminess (N)	140.21 ± 3.53^a^	121.25 ± 1.84^b^	97.80 ± 2.02^c^	85.12 ± 2.99^d^	249.856***
	Cohesiveness (%)	0.96 ± 0.02^a^	0.95 ± 0.01^a^	0.96 ± 0.02^a^	0.93 ± 0.04^a^	1.086

^1^ One‐way ANOVA was used. Different letters in the same row (a–d) demonstrate a significant difference (*p < 0.05, **p < 0.01, ***p < 0.001).

These results indicated that the incorporation of oil disrupted molecular rearrangement and reduced the rate of gelatinized starch chain reassociation, thereby delaying structural hardening. This observation aligns with Lan et al. (2024), who reported that the formation of amylose–lipid complexes inhibits amylose recrystallization, reduces gel firmness, and improves storage stability. Collectively, the findings suggest that 4% oil was the optimal concentration for maintaining desirable texture attributes and inhibiting retrogradation, supporting its use as an effective formulation strategy in starch‐based gel product development.

#### 3.2.2 Syneresis Properties

**FIGURE 3:**
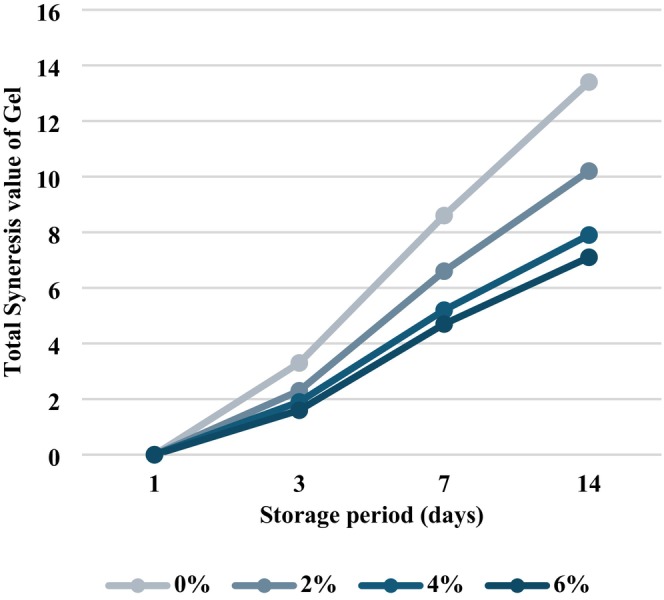
Syneresis rate of whole Apios gel based on oil content during storage. The graph illustrates the syneresis values of whole Apios gel with 0%, 2%, 4%, and 6% oil added, over 1, 3, 7, and 14 days at 4°C.

Syneresis of whole Apios gel containing different oil concentrations (0%, 2%, 4%, and 6%) was evaluated during storage at 4°C on days 1, 3, 7, and 14 (Table 5, Figure 3). No moisture separation was observed on day 1 for any sample. However, by day 3, the syneresis rate differed significantly according to oil concentration (*p* < 0.001), with values of 3.43% ± 0.12% (0% oil), 2.40% ± 0.10% (2% oil), 1.93% ± 0.06% (4% oil), and 1.53% ± 0.06% (6% oil). On day 7, syneresis further increased across all samples, but the 4% and 6% oil groups continued to exhibit considerably lower water separation (3.40% ± 0.10% and 3.00% ± 0.10%, respectively) than the control (5.27% ± 0.06%). After 14 days of storage, syneresis in the control gel reached 4.30% ± 0.20%, whereas the 4% oil sample demonstrated a significantly lower value of 2.73% ± 0.06%, indicating superior water retention capacity (*p* < 0.001).

**TABLE 5:** Syneresis rate of whole Apios gel depending on oil content during storage.

Mean ± SD					
	Oil content (%)				*F*‐value^1^
	0	2	4	6	
Day 1	0.00 ± 0.00^d^ ^1^	0.00 ± 0.00^d^	0.00 ± 0.00^d^	0.00 ± 0.00^d^	—
Day 3	3.43 ± 0.12^Ac^	2.40 ± 0.10^Bc^	1.93 ± 0.06^Cc^	1.53 ± 0.06^Dc^	268.556***
Day 7	5.27 ± 0.06^Aa^	4.17 ± 0.15^Ba^	3.40 ± 0.10^Ca^	3.00 ± 0.10^Da^	255.881***
Day 14	4.30 ± 0.20^Ab^	3.53 ± 0.06^Bb^	2.73 ± 0.06^Cb^	2.33 ± 0.06^Db^	183.000***
*F*‐value	1112.863***	1102.152***	1560.267***	1201.867***	

^1^ One‐way ANOVA was used. Different letters in the same row (a–d) or in the same column (A–D) indicate a significant difference (***p < 0.001).

The consistent decrease in syneresis with increasing oil concentration suggested that oil interfered with starch chain reassociation and reduced structural contraction within the gel matrix. Previous research (Lan et al. 2024) reported that lipid addition promotes water retention in starch gels by forming amylose–lipid complexes that act as barriers to recrystallization, thereby limiting moisture release. Similarly, Hong et al. (2024) demonstrated that reduced intermolecular interactions among starch polymers enhance network stability and diminish water expulsion. In agreement with these findings, the current results confirmed that 4% oil most effectively suppressed moisture separation and improved retrogradation stability.

Overall, the reduction of syneresis with increasing oil concentration highlighted a positive effect of oil on maintaining gel quality during storage, supporting the use of oil incorporation as an effective strategy for improving texture and shelf life performance of whole Apios gel products.

#### 3.2.3 Retrogradation Properties by Differential Scanning Calorimetry (DSC)

Differential scanning calorimetry thermograms (DSC) were measured to investigate the effect of oil addition on the retrogradation of whole Apios gel (Table 6). DSC is a technique that measures the difference in energy input to a substance and a reference substance as a function of temperature, and the ΔH (enthalpy) change value is the area under the peak in the DSC curve, representing the total amount of heat absorbed or released by the sample over a specific temperature interval (Karim et al. 2000). In this study, on day 7, ΔH values decreased from 4.02 J/mg for the control (0% oil) to 2.40 (2% oil), 0.65 (4% oil), and 1.41 J/mg (6% oil), with the lowest value consistently observed in the 4% oil sample. A similar pattern was maintained on day 14, where ΔH increased to 6.01 J/mg in the control but remained substantially lower in the 4% oil sample (0.77 J/mg), indicating significantly reduced recrystallization and enhanced retrogradation inhibition. The onset temperature (To), which is the temperature at the point where the measurement curve begins to rise or fall, was higher at 4% and 6% oil content before 7 days of storage, and higher at 2% and 4% oil content after 7 days. It was believed that before the storage period of 7 days, the addition of more than 4% oil exhibited structural stability, but over time, the retrogradation inhibition effect significantly decreased when the oil content exceeded 4% after 7 days, resulting in a decrease in structural stability and the onset of changes at lower temperatures. Peak temperature (Tp) was marked at the point on the curve where the largest change in temperature occurred. After 3 days of storage, the highest peak temperature was observed at 4% oil content, and the peak temperature tended to decrease when the oil content exceeded 4%. This was attributed to the inhibition or retardation of recrystallization at 4% oil content, resulting in high thermal stability. The completion temperature (Tc), the final temperature at which the curve stabilized again, showed some variation depending on the oil content, with similar trends noted depending on the storage period. The enthalpy changes values increased when the oil content exceeded 4%, thus exhibiting a similar trend as observed for To and Tp. These results demonstrate that moderate oil addition effectively interferes with molecular rearrangement during storage, reducing retrogradation rates. The strong inhibitory effect of 4% oil supports previous findings (Lan et al. 2024), in which amylose–lipid interactions reduced recrystallization and improved thermal stability. Collectively, DSC results confirmed that 4% oil was the optimal concentration for maintaining thermal and structural stability of whole Apios gel during storage. These findings should be interpreted with caution, as the freeze‐drying and rehydration processes required for DSC sample preparation may modify the native gel microstructure and affect thermal transition measurements. Further studies will apply DSC directly to hydrated gel samples to minimize sample alteration and improve evaluation accuracy.

**TABLE 6:** Differential scanning calorimetry (DSC) of whole *Apios* gel retrogradation depending on oil content during storage.

		Oil contents (%)			
		0	2	4	6
Day 1	To (°C)	20.28	45.99	47.89	49.64
	Tp (°C)	59.32	75.57	66.24	78.96
	Tc (°C)	114.88	114.21	115.00	114.37
	ΔH (mJ/mg)	3.01	1.89	0.48	0.87
Day 3	To (°C)	20.86	37.71	43.44	45.84
	Tp (°C)	68.95	74.56	79.62	72.95
	Tc (°C)	114.70	114.59	113.92	113.92
	ΔH (mJ/mg)	3.86	2.04	0.50	1.06
Day 7	To (°C)	21.10	41.36	45.43	20.95
	Tp (°C)	65.53	69.75	71.12	62.18
	Tc (°C)	114.75	115.19	114.24	114.46
	ΔH (mJ/mg)	4.02	2.40	0.65	1.41
Day 14	To (°C)	19.94	54.90	53.10	19.96
	Tp (°C)	57.11	79.50	83.14	65.40
	Tc (°C)	115.12	112.31	114.40	114.80
	ΔH (mJ/mg)	6.01	2.41	0.77	1.82

Abbreviations: ΔH, Enthalpy; Tc, Completion temperature; To, Onset temperature; Tp, Peak temperature.

#### 3.2.4 Retrogradation Properties by Fourier Transform Infrared Spectrophotometer (FTIR)

**FIGURE 4:**
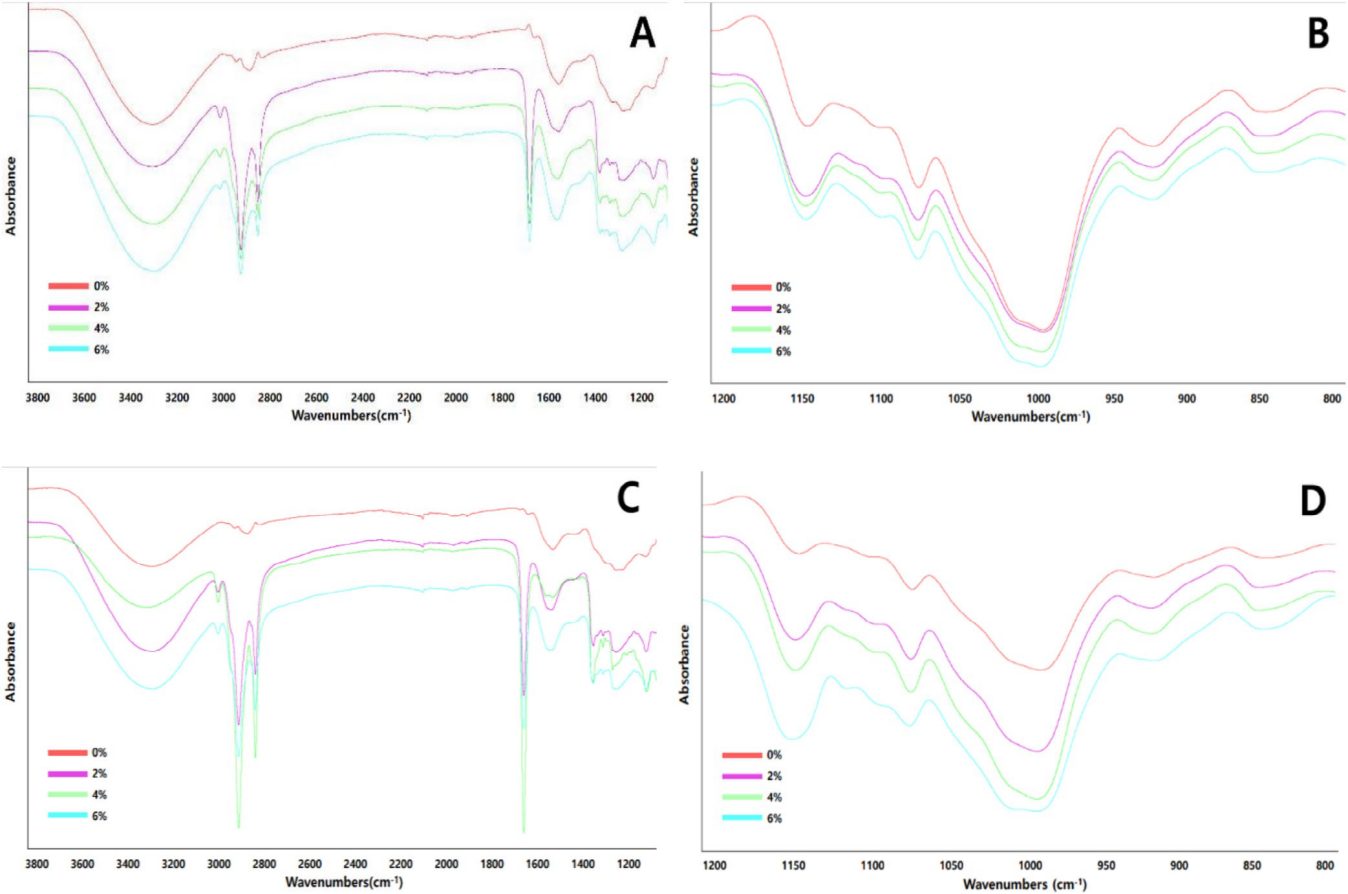
FTIR analysis of whole Apios gel retrogradation based on oil content during storage. FTIR spectra of whole Apios gel without and with added oil are depicted in parts A and B on day 1, while parts C and D represent the spectra on day 14.

FTIR analysis was conducted to explore the impact of oil addition on the retrogradation of whole Apios gel. FTIR is known to relate to the crystallization, molecular chain conformation, and helical structure of starch and is utilized to analyze changes in short‐range aligned structures (Zou et al. 2020). Table 7 and Figure 4 illustrate the various FTIR spectra of whole Apios gel influenced by oil.

**TABLE 7:** Fourier transform infrared spectrophotometer (FTIR) analysis of whole *Apios* gel retrogradation based on oil content during storage.

Sample		Oil contents (%)			
		0	2	4	6
3280 cm^−1^ ^1^	Day 1	3.053	3.048	3.045	3.040
	Day 14	3.052	3.048	3.036	3.031
2923 cm^−1^	Day 1	—	3.423	3.421	3.421
	Day 14	—	3.422	3.421	3.420
2852 cm^−1^	Day 1	—	3.870	3.869	3.869
	Day 14	—	3.871	3.870	3.871
1740 cm^−1^	Day 1	—	5.734	5.734	5.735
	Day 14	—	5.734	5.735	5.735
1152 cm^−1^	Day 1	8.717	8.713	8.704	8.689
	Day 14	8.705	8.704	8.698	8.684
1077 cm^−1^	Day 1	9.290	9.278	9.282	9.283
	Day 14	9.256	9.280	9.282	9.284
995 cm^−1^	Day 1	10.036	10.032	10.024	10.014
	Day 14	10.042	10.034	10.026	10.023

^1^ Cm^−1^ refers to the wavenumber, defined as the reciprocal of the wavelength.

The spectra in the range of 3700–3100 cm^−1^ impacted the vibrations of OH groups, associated with hydroxyl group bonding, particularly hydrogen bonding (Barragán‐Martínez, Molina‐Rodríguez, et al. 2022). This experiment demonstrated that the intensity of hydrogen bonding at the 3280 cm^−1^ peak was notably weaker in the sample with oil than in the sample without oil. A previous study examining structural changes due to emulsion addition (Huang et al. 2024) identified two peaks within the 3600–3100 cm^−1^ range, where reduced intensity indicated weakened hydrogen bonds, correlating with the inclusion of an oil‐containing emulsion. The results of this experiment were consistent with those of the current study, suggesting that lipid complex formation interfered with hydrogen bonding and contributed to retrogradation inhibition.

The peaks at 2923 and 2852 cm^−1^ in the FTIR spectrum corresponded to CH stretching vibrations associated with starch–lipid complexes (Barragán‐Martínez, Román‐Guerrero, et al. 2022). A previous study (Lan et al. 2024) identified similar peaks in soybean oil‐added samples compared to the control, attributed to lipid binding to the amylose helix through hydrophobic interactions. The peak around 1740 cm^−1^ was attributable to carbonyl (C=O) group vibration, and its presence in all oil‐added gel samples indicated that oil incorporation influenced the FTIR spectrum (Wang et al. 2019).

Structural FTIR spectral features of starch appeared in the 800–1300 cm^−1^ region (Xiong et al. 2017). The peak around 1152 cm^−1^, associated with C–O–C and C–OH bonds, remained relatively unchanged with storage time in oil‐added gels, indicating stabilization (Pozo et al. 2018). The peak at 1077 cm^−1^, linked to amylose and amylopectin backbone structures, remained relatively constant in oil‐added gels but increased in the control gel during storage.

The peak around 995 cm^−1^ was associated with intramolecular hydrogen bonding and the degree of molecular rearrangement during retrogradation (Lan et al. 2024). Although peak intensity increased with storage time in all samples, gels containing 2% and 4% oil exhibited smaller changes than the control, indicating effective retrogradation inhibition. Overall, FTIR analysis confirmed that oil weakened hydrogen bonding and reduced short‐range ordering associated with retrogradation, with moderate oil concentrations showing the most stable structural patterns.

## 4 Conclusions

This study evaluated the feasibility of developing a whole Apios gel using the entire tuber and compared its quality and retrogradation characteristics with Apios starch gel. Whole Apios gel demonstrated significantly lower syneresis and superior sensory acceptance compared with starch gel, indicating advantages in water retention and consumer preference. In addition, the incorporation of oil (0%–6%) effectively inhibited retrogradation, with 4% oil showing the strongest effect, as evidenced by reduced hardness and gumminess, lower syneresis values, and the lowest enthalpy change in DSC analysis. FTIR spectra further confirmed weakened hydrogen bonding and suppressed short‐range crystallinity in oil‐added samples, supporting the inhibitory role of oil on starch molecular reassociation.

These results demonstrate that whole Apios gel offers nutritional, functional, and sustainability benefits by utilizing the entire tuber without starch extraction, and that 4% oil is the optimal concentration for improving textural stability and delaying retrogradation during storage. The findings support the potential industrial application of whole Apios gel as a novel gel‐based food product. Future research should investigate processing scale‐up and the influence of storage environments, such as temperature and humidity, to enhance commercialization potential.

## Author Contributions

**Nami Joo:** project administration, supervision, writing – review and editing. **Soo Hyun Kim:** data curation, resources, writing – original draft, methodology, investigation, visualization, validation, conceptualization, formal analysis. **Ju Hong Park:** data interpretation and critical revision of the manuscript.

## Funding

This research was supported by a grant of the FoodTech RnD Center Development and Support Program through the Gyeongbuk Technopark (GBTP) funded by GYEONGSANGBUK‐DO and Pohang City (GBTP2023129001); by a grant of the High Value‐added Food Technology Development Program through the Korea Institute of Planning and Evaluation for Technology in Food, Agriculture and Forestry (IPET) funded by the Ministry of Agriculture, Food and Rural Affairs (MAFRA, RS‐2024‐00403998 and RS‐2024‐00403987); and by a grant of the Agriculture and Food Convergence Technologies Program for Research Manpower Development through IPET, funded by MAFRA (RS‐2024‐00402136).

## Data Availability

The data that support the findings of this study are available from the corresponding author upon reasonable request.
